# Multifunctional Cement Mortars Enhanced with Graphene Nanoplatelets and Carbon Nanotubes

**DOI:** 10.3390/s21030933

**Published:** 2021-01-30

**Authors:** Panagiota T. Dalla, Ilias K. Tragazikis, George Trakakis, Costas Galiotis, Konstantinos G. Dassios, Theodore E. Matikas

**Affiliations:** 1 Department of Materials Science and Engineering, University of Ioannina, 45110 Ioannina, Greece; p.dalla@uoi.gr (P.T.D.); i.tragazikis@uoi.gr (I.K.T.); matikas@uoi.gr (T.E.M.); 2 Institute of Chemical Engineering Sciences, Foundation of Research and Technology-Hellas, (FORTH/ICE-HT), Stadiou Street, Platani, 26504 Patras, Greece; trakakis@iceht.forth.gr (G.T.); galiotis@chemeng.upatras.gr (C.G.); 3 Department of Chemical Engineering, Caratheodory 1, University of Patras, 26504 Patras, Greece

**Keywords:** cement mortars, graphene nanoplatelets, multi-wall carbon nanotubes, mechanical properties, fracture toughness, acoustic emission, electrical conductivity

## Abstract

Recent findings have brought forward the potential of carbon nano-species, especially nanotubes and graphene, to impart exceptional multifunctional potential to cement, offering simultaneous enhancement of mechanical, fracture mechanical and electrical properties. While available knowledge on the topic is still limited, there is a complete absence of direct comparisons of the potential of the nano-species to improve strength and toughness and provide multifunctionality to the mortars. The study offers a comprehensive overview of these potentials, for mortars modified with pure graphene nanoplatelets and carbon nanotubes at consistent, directly comparable, concentrations up to 1.2 wt.%. Testing included flexure under pure bending moments, axial compression, electrical resistivity measurements and fracture tests under three point bending configuration; the latter were also independently assessed by acoustic emission. Differences in documented properties and optimal concentrations associated with improved mechanical performance were directly compared and rationalized in terms of nanospecies morphology. Dramatic, statistically consistent improvements in fracture behavior, up to 10-fold of control values, were documented for specific nanofiller concentrations, indicating an excellent potential of the material system for contemporary smart construction applications. An exceptionally favorable comparison of acoustic emission and fracture energy data confirmed that the non-destructive technique can independently assess the fracture performance of mortars with exceptional precision.

## 1. Introduction

As the major component of the popular construction material, cement is constantly being improved to meet the requirements of increasingly complex structures [1]. Significant scientific effort has been invested in the improvement of its inherently low tensile strength, low fracture toughness and brittleness, by the embedment of reinforcing phases. While ductility and resistance to tensile stresses are usually imparted by the introduction of steel rods in concrete and other cementitious materials, additional reinforcement can be achieved by the inclusion of micron-scale materials [2,3,4,5]. As already established [6,7,8,9], glass, aramid, polypropylene and basalt fiber additives can inhibit crack growth and propagation and thus control the failure modes of concrete composites. To meet the need for increasingly safe and complex structures, contemporary applications demand not only strong and tough, but also multifunctional and smart cement-based material systems, with sensing capabilities and good combination of mechanical, electrical and thermal properties [10,11,12]. Smart cementitious materials with enhanced electromechanical properties can help the development of buildings and infrastructures with integrated sensing and health-monitoring capabilities, thus increasing both the structural safety and service life of reinforced concrete structures [13,14].

To that end, dispersion in the concrete bulk of nanoscale materials such as carbon nanotubes (CNT), the most important and readily available form of 1D nano carbon allotrope and, lately, graphene, the revolutionary planar sheet of atomic thickness constructed of sp^2^ carbon atoms, has lately been suggested as a potential new strategy for the improvement of the mechanical, electrical and damage sensing behavior of concrete [5,15]. In fact, such nano-carbon are currently regarded as ideal candidates for imparting sensing abilities, hence also smartness and multifunctionality to a whole range of composites for a wide range of applications such as in the fields of materials engineering, electronics and aerospace [4,16,17,18,19]. At the same time, extensive analytical research has been focused on the investigation of the nano-fillers’ effects in nanocomposite materials [20,21,22,23]. CNTs are most commonly produced by chemical vapor deposition (CVD) and their diameters range from 0.4 nm (single-walled tubes, SWCNT) to couple tens of nm (multi-walled tens, MWCNTs). Due to their commercial availability at low prices, the latter constitute the more popular and affordable choice, albeit of slight inferior performance to pricier SWCNTs. Owing to its low fabrication rates and high costs, pure graphene is not yet mass-produced. Graphene Nanoplatelets (GNP), on the other hand, composed of few layer graphene and usually obtained through liquid phase exfoliation and centrifugation, combine large-scale production and low costs with the remarkable physical properties of graphene and those of graphite [24]. Their planar geometry, offering higher contact area than CNTs, has been suggested to provide more sites for physical or chemical attachment to the continuous host phase, leading to superior reinforcement potential, compared to the one-dimensional tubes [25].

In the fundamentals of reinforcement, the key to efficient transfer of load to the reinforcing phase is the establishment of a homogeneous dispersion of the latter within the **continuous phase**. Homogenization of aqueous suspensions of carbon nanospecies by ultrasonication usually requires the use of surfactants, which facilitate the dispersion of the otherwise hydrophobic species in the aqueous environment. However, the addition of such compounds does not come without side-effects. Yazdanbakhsh et al. [26] reported on the incompatibility between dispersion-aiding surfactants and cement during the hydration phase, while Makar et al. [27] observed a reduction in the MWCNTs’ aspect ratio due to their exposure to the high ultrasonication energy densities used for efficient disentanglement of the tubes. The finding signified that the desirable tube separation observed in the parent aqueous suspension is not maintained in the resulting cement environment. 

The characterization of the mechanical, electrical and thermal performance of mortars with CNT and GNP reinforcements has been the focus of several studies in the literature [13,14,28,29,30,31,32,33,34,35,36,37,38]. Mortar permeability was found to depend on the nano-inclusions due to the increase in absorbed ion chlorides with nanotube loading [36]. Siddique and Mehta [28] studied the effect of CNTs as fillers on fly ash cement mixes and found both compressive and flexural strengths to increase with respect to plain specimens and specimens reinforced with carbon fibers. In addition, cement-based materials with varying amounts of carbon nanotubes, carbon black, carbon fibers and graphene nanoplatelets exhibited excellent piezoresistive performance during in situ capturing of damage evolution in specimens loaded in compression [5,13,35,39]. In their study on the effect of surfactant-coated graphene nanoplatelets (GNPs) in notched-beam cement mortars, Zohhadi et al. reported increases in the flexural strength and elastic stiffness of modified cement paste as well as on mortar compressive strength [40]. Despite the amount of scientific effort invested in the field, especially in CNT-enhanced systems, a systematic comparison of the mechanical properties of cement–based mortars enhanced with carbon nanotubes and graphene nanoplatelets, at directly comparable nanofiller concentrations, is currently unavailable. Additional difficulty rises from the fact that different laboratories use incomparable nano-filler concentrations and experimental protocols.

Moreover, extremely limited information about the fracture properties of CNT- and especially graphene-enhanced cement-based materials is available in the literature. Mortars and geopolymer concrete with additives such as limestone and fly ash/metakaolin have been studied [41,42,43], while Stynoski et al. [44] reported on the fracture behavior of notched Portland cement mortars reinforced with 0.855 wt.% of cement carbon fibers, 0.125 wt.% of cement CNTs and silica fume. Documented improvements of 35 and 56% in toughness and critical crack tip opening displacement (CTODc), respectively, over the control duration of 28 days were attributed to the simultaneous presence of silica fume and carbon nanotubes in the parent mixture. The net effect of carbon nanotubes in fracture toughness and CTODc was quantified at 10% and 20% improvement, respectively, within the same control period. In [45], improvements in the fracture energy of cement were documented for a GNP concentration of 0.4 wt.%. To the authors’ knowledge, the recent work by Liu et al. [46] on the improvement of fracture toughness of CNT/graphene cement paste specimens, although it does not concern mortars, is the closest available approach on the topic vis-à-vis the work reported here. The self-sensing properties of cement mortars with embedded conductive nanofillers such as carbon nanotubes, graphene nanoplatelets and carbon nanofibers has been the topic of several literature studies [5,47]. Previous research has also brought forward the promising structural health monitoring capabilities of conductive nanofiller-doped cement-based materials, mainly by the application of experimental methods such as by electrical resistance and biphasic DC measurements, and data-driven approaches [48,49,50].

Assessing the structural integrity of concrete structures by non-destructive techniques is of paramount importance towards understanding and optimizing their performance [51]. Acoustic emission (AE), relying on the collection of energy signals, rapidly released from various locations in a material, is one of the most promising techniques towards that end. The generated elastic waves travel through the material and the released acoustic energy is indicative of the extent and severity of the damage therein [52]. AE can detect crack initiation and growth, as well as fatigue and corrosion damage in a wide range of materials like concrete and composites while the waveform shape provides information on the material’s mode of fracture [45,53,54,55]. On the other hand, electrical measurements have been utilized to monitor moisture displacement inside cement-based materials, assess the structural integrity relating to the level of inflicted damage, perform in-depth investigations of changes in the microstructure of cementitious materials and healing by microcrack closing [56,57,58]. While studies appear to adopt custom testing protocols, it is now well established that the four-point probe electrical resistivity measurement method is preferable over is two-point counterpart as it offers the ability of separating true resistivity from measured resistivity, which the latter does not offer [59,60,61].

The current paper reports the results of a systematic comparative analysis of the mechanical, fracture mechanical and electrical potential of cement mortars enhanced with pure graphene nanoplatelets and carbon nanotubes at directly comparable concentrations. Variations in documented behavior are discussed in view of nanocarbon morphology while critical concentrations associated with optimal performance are reported and compared. The dramatic effect of CNT and GNP presence in the improvement of the fracture behavior of the materials is highlighted. The potential of acoustic emission as an independent, non-destructive tool for assessing the fracture performance of mortars is discussed herein.

## 2. Materials and Methods

### 2.1. Materials and Specimens

The fillers chosen for the present study were nanocarbons of different dimensionalities and structures; they were anticipated to enhance both mechanical and−as conductive matter−also the electrical properties of the materials. The catalytic CVD-prone, multi-walled carbon nanotubes utilized herein were one-dimensional concentric cylinders acquired by Nanotech Port Co. Ltd., Shenzhen, China; their properties are presented in Table 1. Commercially available pristine graphene nanoplatelets, Pure Graphene PlusTM, were acquired in dry powder form by Directa Plus SpA, Lomazzo, Italy; they were two-dimensional stacks of graphene layers; their nominal thickness of 2–3 nm indicates the presence of 5 to 7 layers of graphene and their properties are presented in Table 2. Two types of mortars were prepared, each modified with one type of carbon nanospecies. The aqueous polycarboxylate polymer-based superplasticizer, Viscocrete Ultra 300 (Sika AG, Baar, Switzerland), which is popular for its chemical stability and for inhibiting air entrapment inside the material, doubled as a dispersion assisting agent. The native cement additive has been proven to enable the dispersion of hydrophic carbon nanospecies in water, providing high levels of homogeneity while rendering the usage of surfactants or functionalization unnecessary [62]. It should be noted that the use of superplasticizers as dispersion agents for carbon nanospecies is not a generic rule and should not be followed without prior experimental proof. 

**Figure 1:**
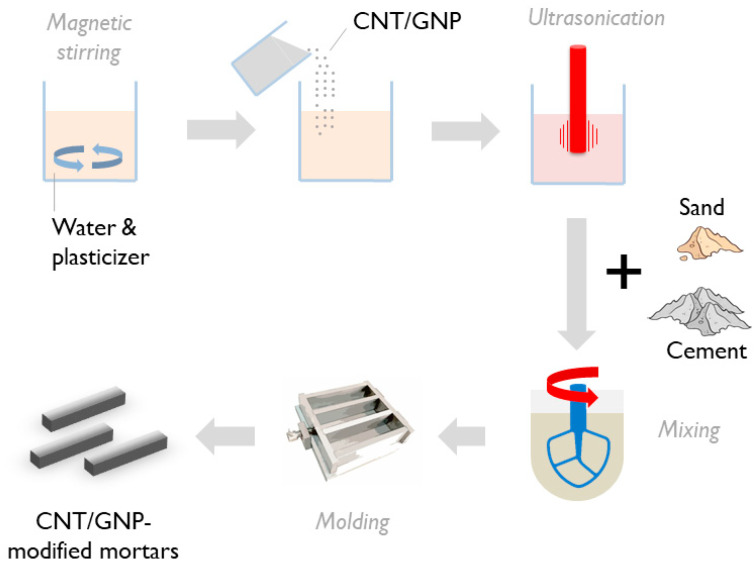
Schematic of experimental protocol adopted for synthesis of carbon nanotubes (CNT)- and GNP-modified mortars.

**Figure 2:**
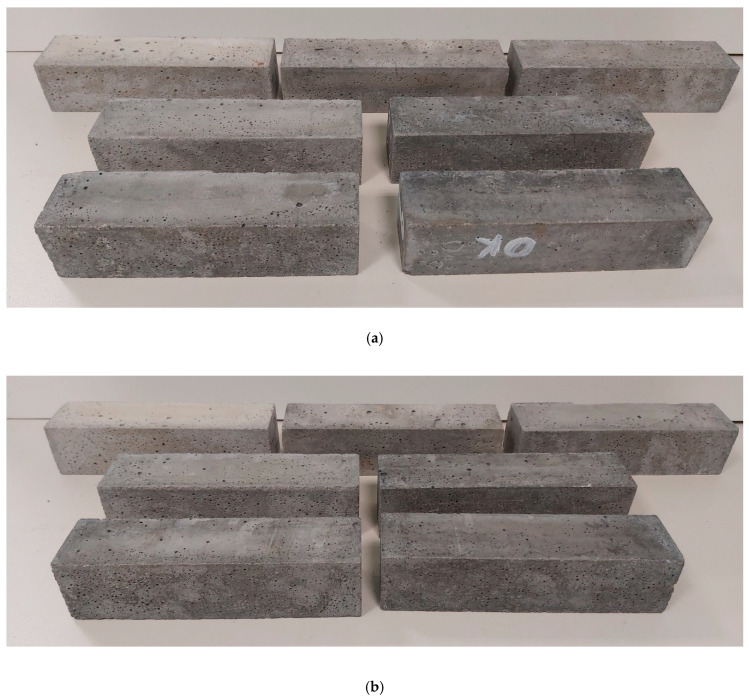
Sets of as prepared CNT- (**a**) and GNP-modified (**b**) mortars at different concentrations. The degree of nano-inclusion concentration is perceived as varying level of darkness of the actual mortar.

For the development of nanomodified mortars with nanotube loadings varying from 0.2 to 1.2 wt.% of cement at a step of 0.2 wt.%, the following experimental protocol was adopted and illustrated in Figure 1. Initially, the tubes and superplasticer were mixed in regular tap water at a mass ratio of 1/1.5 and then magnetically stirred for a duration of 2 min. These suspensions were rendered homogeneous by exposure for 90 min to a sonication power of 4500 J/min. For this task, a Hielscher Ultrasonics UP400S tip-ultrasonicator (Hielscher GmbH, Teltow, Germany) was used, the sonotrode diameter was Ø22 mm and the frequency was kept constant at 24 kHz. This combination of ultrasonication parameters has been experimentally determined as optimum for the establishment of homogeneity without CNT aspect ratio impairment [63]. After further processing in a vacuum chamber for degassing, resultant suspensions were transferred in a rotary mixer and mixed with ordinary Portland cement (OPC type I-42.5N) and natural sand for a duration of 4 min, following BS EN 196-1 recommendations. In all mixtures, water-to-cement ratio remained constant at 1/2. To allow direct comparison, the same mixing protocol was adopted for the preparation of mortars enhanced with graphene nanoplatelets at loadings ranging from 0.2 to 1.2 wt.% of cement at a step of 0.2 wt.%. In total, six specimens were produced at each percentage of each of the two nano-inclusions. After the end of the mixing phase, fresh mortars were transferred into oiled metallic forms, dimensions of 160 mm × 40 mm × 40 mm, where they were allowed to rest for 24 h. After demolding, the resultant prisms were left to mature in a 100% humidity environment for the standard duration of 28 days. Control mixtures free of nano-inclusions were also processed for reference purposes. Photographs of two typical sets of final specimens, each for every nano-inclusion used, are presented in Figure 2. After the end of the maturing period, 20 mm-deep initial notches were machined in 3 specimens of each nano-filler concentration.

### 2.2. Testing Methods

#### 2.2.1. Bending and Compressive Test

Four point bending tests were performed as per ASTM C1609 guidelines for characterization of the bending strength under pure bending moments. The tests were conducted on a 30 kN testing frame (Instron 5967, Instron, Norwood, MA, USA) for all types of mortars. Compressive strength characterization was performed on the two failed halves of these tests prisms as per EN 196-1:2005 recommendations. The compressive load was applied at a rate rate of 2400 N/s, corresponding to a stress rate of 1.5 MPa/s.

#### 2.2.2. Fracture Toughness

**Figure 3:**
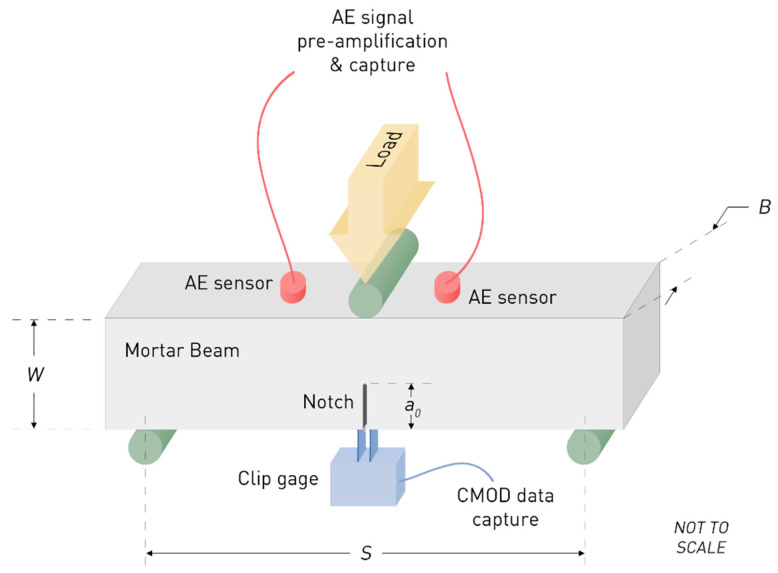
Three-point bend testing configuration with in situ acoustic emission monitoring.

The mortar’s fracture behavior was characterized through three point bending testing with in situ monitoring of acoustic emission response of the material; a schematic of the adopted experimental arrangement is presented in Figure 3. To enable attainment of maximum load within the first 60 s of the test duration, a crosshead displacement of 0.01 mm/s was employed. A knife-edge-mounted Instron^TM^ (Instron, Norwood, MA, USA) crack mouth opening displacement (CMOD) gage, gauge length of 10 mm was used to measure the instantaneous CMOD. Knife-edge thickness, *d*, was accounted for in the calculations as explained in the following paragraph.

The fracture toughness of nanomodified mortars was calculated from the Hilleborg linear elastic fracture mechanics crack model [64], which is equivalent to the Dugdale-Barenblatt model for metals. Therein, *CMOD* for the notched beam geometry loaded at the center-point with an applied load *P*, is given by [65]:

(1) $$CMOD=\frac{6PSa}{EBW^{2}}g(\alpha )$$

where *S* is the span of the beam, *a* the crack length, *g*(*α*) a dimensionless geometry function, *α* = *a*/*W* the ratio of crack length over specimen width *W*, *E* is the Young’s modulus of the material and *B* specimen thickness. In the present study, the span, *S*, was *S* = 3 *W* while *α*_0_ = a_0_/*W*, were a_0_ the initial notch length, was 0.25. The values of *g(α)*, are calculated through [65]:

(2) $$g(\alpha )=g_{1}(\alpha )+g_{2}(\alpha )$$

where

(3) $$g_{1}(\alpha )=\frac{0.995-0.547\alpha }{1-2.06\alpha +1.063\alpha^{2}}$$

(4) $$g_{2}(a)=0.342-1.761\alpha +2.098\alpha^{2}-1.437\alpha^{3}+1.455\alpha^{4},\;\mathrm{for}\;0.05\leq a\leq 0.80$$

or

(5) $$g_{2}(\alpha )=0,\;\mathrm{for}\;0.80\leq \alpha \leq 0.90$$

As in most three point bending tests, *CMOD* is herein measured at a distance *d*, equal to the knife edge thickness, from the notch mouth. Hence, determination of the true *CMOD* value requires correction of the measured value, *CMOD_measured_*, for that thickness [65]. Thereafter,

(6) $$CMOD=k_{d}CMOD_{measured}$$

where *k_d_* is a conversion factor which, for *d* between 1 and 6 mm, can be calculated from

(7) $$k_{d}(d,a)=\frac{h_{1}+h_{2}a}{1+h_{3}a+h_{4}a^{2}}$$

The values of coefficients *h_i_*, for d = 3 mm, are given in Table 3.

#### 2.2.3. Fracture Toughness

Fracture energy, which is the energy required for the creation of a crack of unit area (SI units of N/m or J/m^2^), can be calculated by [65]:

(8) $$G_{F}=\frac{W_{0}+mg\delta_{0}}{A_{lig}}$$

where *W*_0_ is the integral of the *P-CMOD* curve, *g* the acceleration of gravity, *δ*_0_ the CMOD at fracture, *A_lig_* the area of the intact ligament and *m* the sum of masses *m*_1_ and *m*_2_, the former being the mass of the specimen and the latter being the mass of the loading device (which does not touch the testing machine but always acts on the specimen):

(9) $$m=m_{1}+m_{2}$$

#### 2.2.4. Acoustic Emission Monitoring

**Figure 4:**
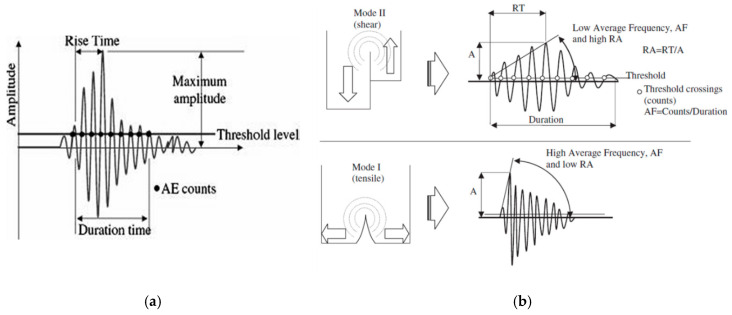
(**a**) Definition of major parameters in an acoustic emission (AE) waveform, (**b**) Types of AE waveforms relating to different fracture modes [66].

The major parameters used in the AE measurements are defined in the acoustic emission waveform scheme presented in Figure 4. Therein, amplitude (*A*) is the maximum voltage of the AE signal measured in decibels (dB); threshold is a user-defined amplitude value in dB based on background noise level; rise time (*RT*) is the time between the first threshold crossing and maximum peak amplitude; *RA* is a key AE parameter defined as the ratio of rise time over amplitude (RT/A); AE counts are the number of times an AE burst crosses the threshold; AE duration is the time between the first and last threshold crossing and AE energy is the area under the amplitude–time curve above the threshold value.

For in situ AE monitoring during three point bending testing of mortars, two resonant broadband 50–400 kHz R15a AE sensors were attached on the un-notched faces of the beams as shown in Figure 3. The sensors, placed at a distance of 40 mm, exhibited maximum sensitivity at 150 kHz and were connected to a Physical Acoustics Corp. PCI-2 board used for waveform collection. The gain of the pre-amplifier was fixed at 40 dB. A threshold of 45 dB was chosen for avoiding noise capture.

#### 2.2.5. Electrical Measurements

**Figure 5:**
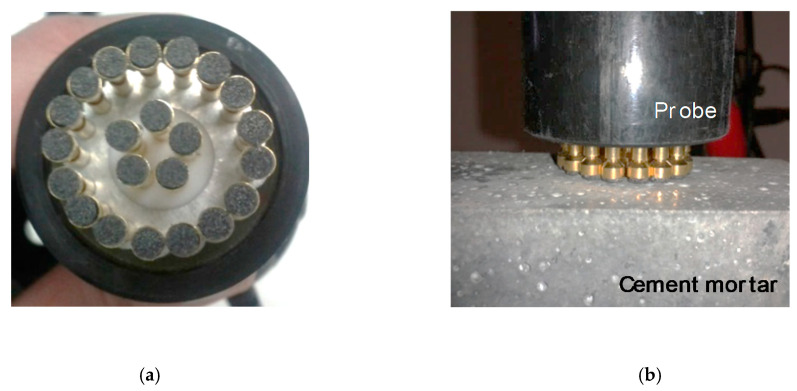
(**a**) Pin electrode arrangement in head of custom electrical probe and (**b**) Sample and probe during resistivity measurement.

DC electrical conductivity measurements were undertaken on the surface of the mortars, by aid of a custom-developed multi-contact circular electrical probe head consisting of 22 concentric circular pin electrodes, as demonstrated in Figure 5a. To allow for maximum contact with the uneven mortar surface, the pins were spring-loaded and had conductive rubber pads attached to their ends. The head was connected to a digital electrometer (Keithley 6517B, Tektronix Inc., Beaverton, OR, USA) with a resistance measurement capacity up to 1018 Ω and an ultra-high current resolution of 10 × 10^−18^ A. The probe head was mounted on a vertical translation stage which could be lowered manually by means of a hand lever to be brought in contact with the mortar surface under investigation (Figure 5b). The mortars, placed below the probe, were resting on an electrically insulating surface to prevent electrical leakage. Surface electrical conductivity was calculated through Ohm’s law, from the measured current at the mortar surface resulting from a known applied voltage value. A total of six samples were probed for each concentration and the average conductivity value of eight consecutive readings in each specimen was calculated and reported.

## 3. Results and Discussion

### 3.1. Bending and Compressive Strength

**Figure 6:**
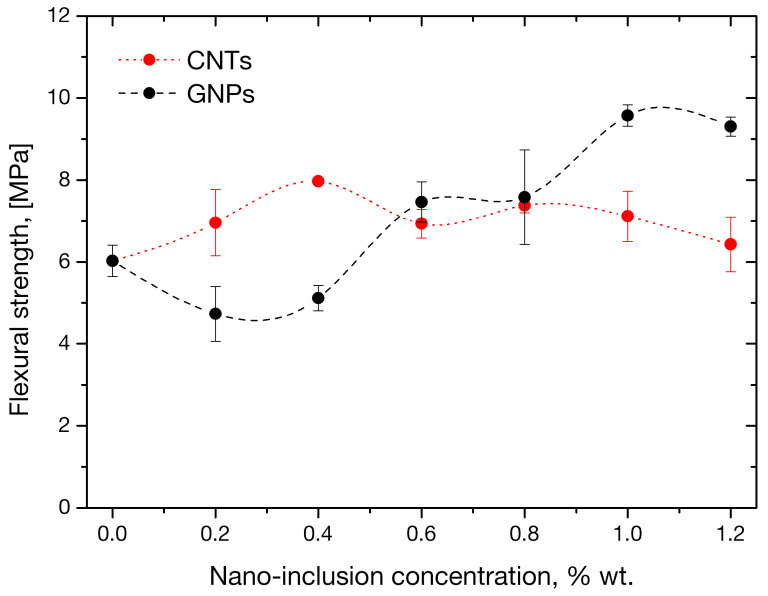
Flexural strength variation in mortars as function of CNT and GNP concentration.

Average values and standard deviations of flexural and compressive strengths of mortars tested as per 2.2.1 are presented in tabular form in Table 4 at each nanofiller concentration and plotted in Figure 6. The maximum improvement in MWCNT-enhanced mortars’ flexural strength, 32% higher than the plain specimens, was observed at a tube concentration of 0.4 wt.%. Flexural strength decreased at higher CNT concentrations, nonetheless remaining higher than the control value; the effect can be attributed to partial CNT entanglement which introduces stress concentration locations. Strength improvements, compared to reference specimens, at nanotube loadings of 0.2, 0.6, 0.8, 1 and 1.2 wt.% were 15, 15, 22, 18 and 7%, respectively.

Concerning GNP effects on flexural strength, low platelet concentrations in the mortars appeared to be linked with decreased flexural performance under pure bending moments, with strength values of mortars of 0.2 and 0.4 wt.% GNP appearing decreased by 21% and 15%, respectively, compared to control specimens. This phenomenon can be explained upon GNP morphology. The nanoplatelets have flake shapes as a result of the stacking of carbon lattice layers. During homogenization, the platelets are distributed throughout the continuous matrix (mortar) at random orientations, and contribute to reinforcement by taking up part of the externally applied load. In absence of the shear component under the pure bending moments of the four point bending configuration, full reinforcement is offered only by the population of GNPs parallel on the neutral plane of the beam along which normal stresses develop. Due to the stochasticity of the phenomenon, this population is of course a small fraction of the total GNPs. At low nanoplatelet concentrations, the absolute number of flakes contributing to reinforcement is practically very small. On the other hand, the contribution of the remaining GNPs to axial reinforcement decreases with an increasing angle of orientation with respect to the neutral plane. At the other extreme, GNPs lying perpendicular to the plane offer the minimum reinforcement, only taking up part transverse load arising solely due to the Poisson effect. As local vertical discontinuities in the material, such perpendicular GNPs can impede the load transfer along the beam axis direction. This effect becomes more important by considering that the interphase between the coarse matrix and the fine nanofiller is expected to be weak. At low GNP concentrations, the small population of reinforcing GNPs is much smaller than load-interrupting ones, hence the observed decreased strength under pure bending moments. At higher concentrations, the axial reinforcement offered by the larger population of fully-reinforcing flakes takes over and strength increases. At GNP loadings of 0.6, 0.8, 1 and 1.2 wt.% there is an improvement of flexural strength by 23, 26, 59 and 54%, respectively.

**Figure 7:**
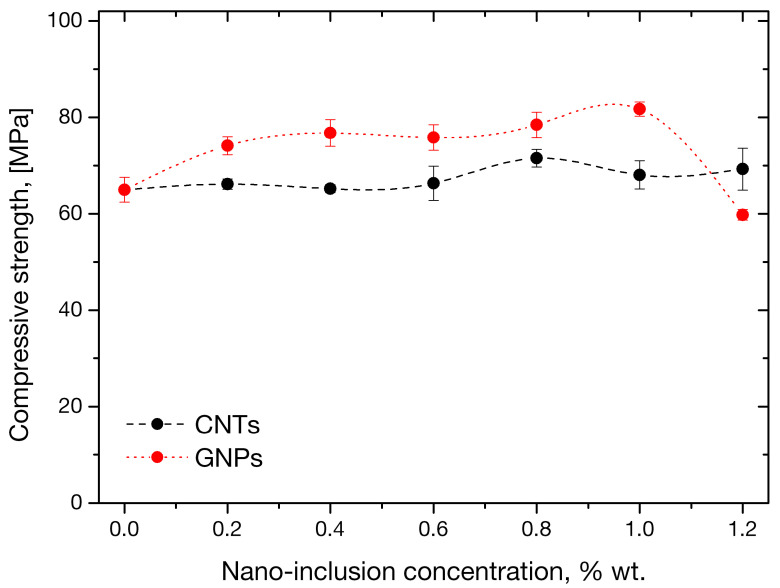
Compressive strength variation in mortars as function of CNT and GNP concentration.

The variation of compressive strength is plotted versus nano-filler concentration in Figure 7. It is therein observed that the property remains practically unaffected by CNT presence within the investigated range of concentration. The maximum improvement in compressive strength was observed at 0.8 wt.% and was of the order of 10%. Graphene nanoplatelet presence appeared to have a better effect on mortar compressive strength; an increase of 26% with respect to the control value was observed at a 1 wt.% GNP concentration with a dramatic decline thereafter. Interestingly, this concentration coincides with the one associated with optimum, albeit limited, improvement in flexural strength by GNPs. This may indicate that the reinforcement offered by the particular nanofiller is efficient under both axial and shear loadings.

### 3.2. Fracture Behavior with in Situ AE Monitoring

**Figure 8:**
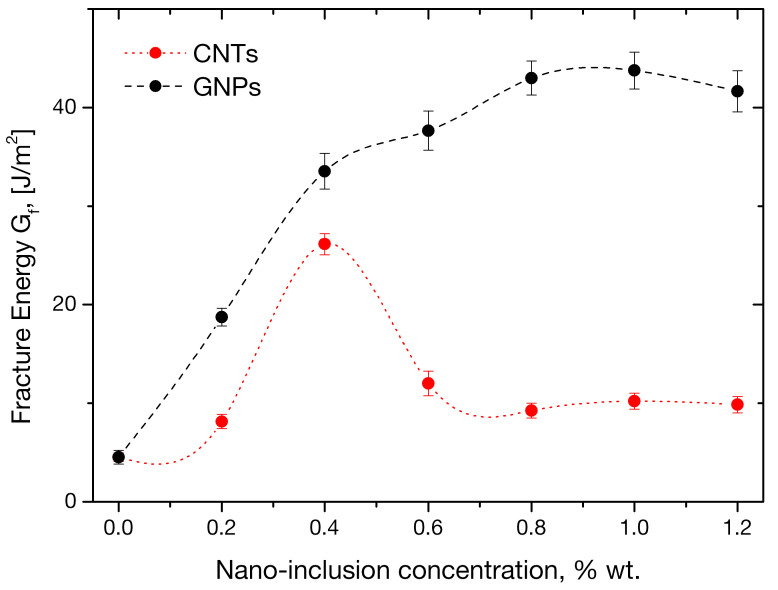
Average fracture energy values during flexural testing of mortars with variable nanofiller concentrations.

Average values and standard deviations of fracture energy, GF, calculated as per 2.2.3, are plotted in Figure 8 and tabulated in Table 5, for mortars with different concentrations of carbon nanotubes and graphene nanoplatelets. It is observed that carbon nanotube presence is related to increased fracture energies, while the tube loading versus GF relation is not monotonic. The maximum increase in fracture energy, a remarkable 479% from the control value, was documented at a CNT concentration of 0.4 wt.%. Above that concentration, the property decreased, nonetheless remaining considerably higher than the control value. It must be emphasized that this is the same tube concentration previously found associated with optimum flexural strength of CNT-enhanced mortars. The fracture energies of nanocomposites with CNT concentrations of 0.6, 0.8, 1 and 1.2 wt.% were found to higher by 166, 118, 105, 126 and 118%, respectively, compared to control specimens.

While the almost 5-fold improvement in fracture energy of mortars loaded with carbon nanotubes at 0.4 weight percent of cement is most notable, the improvement of the property in graphene-enhanced mortars is even more dramatic. Percentile improvements of 315, 643, 735, 853, 870 and 823% from the control value for respective GNP concentrations of 0.2, 0.4, 0.6, 0.8, 1.0 and 1.2 wt.% are reported. It is interesting to highlight the small standard deviation associated with the measurements, of the order of 5% of average values, indicating high repeatability. Comparing the fracture energies of mortars at the same concentration of nano-fillers, graphene-related ones appear higher by 292, 34, 342, 712, 590 and 595%, for loadings of 0.2, 0.4, 0.6, 0.8, 1 and 1.2%, respectively. Based on these findings, a definite distinction of the 0.4% carbon nanotube loading as optimal for imparting overall superior mechanical performance and especially dramatic improvement of the mortars’ fracture resistance can be concluded. On the other hand, while the behavior of the fracture energy of mortars enhanced with graphene nanoplatelets follows a rather smoother growth, without sharp peaks as in the case of nanotube-enhanced mortars, a clear distinction of 1wt.% GNP loading as optimal for both mechanical and fracture mechanical performance improvement appears relevant. It is interesting to investigate the origins of the almost monotonic increase in fracture energy in GNP-modified mortars in contrast to the well-established peak-type improvement offered by CNTs. The authors conceive the effect as a combination of the filler geometry and type of loading. CNTs are rod-like structures which, in the absence of any external stimulus, are expected to lie wavy in the cement’s mix water, hence being prone to entanglement due to mechanical interlocking. Such entanglements act as stress concentrators and discontinuities in the final CNT-modified material, limiting its strength and fracture toughness. GNPs are plane nano-reinforcements which not only do not entangle by mechanical interlocking, but also exhibit exceptional mechanical response under shear loading due to very good dry sliding properties between the individual graphene layers in the platelets. They are hence expected to outperform the tubes in shear loading scenarios such as three-point bending and offer monotonically increasing improvement up to higher concentrations, as observed herein. The reported dramatic improvements in fracture energy are not only highly desirable for an otherwise inherently brittle material system such as cement, but also unfold possibilities for the design of next level cement-based construction systems.

**Figure 9:**
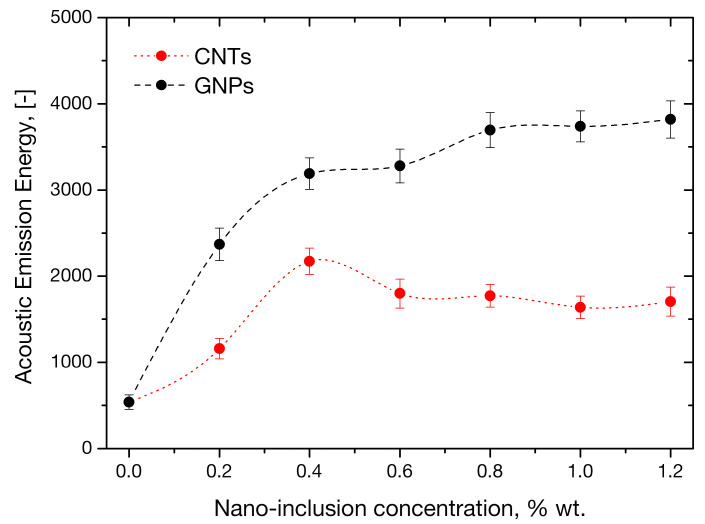
Average AE energy values during flexural testing of mortars with variable nanofiller concentrations.

AE results offered an independent evaluation of the materials’ mechanical and fracture mechanical performance. It must be remembered that AE energy, as a non-mechanical property, is not directly related to flexural strength; however it is directly related to the energy released during flexural loading which, in turn, relates to fracture toughness [51,52,53]. The cumulative AE energy captured in situ during bending of the nano-reinforced mortars is plotted as versus nanofiller concentration in Figure 9. Impressively, the AE energy trend with both CNT and GNP concentrations was found almost identical to the corresponding trends of fracture energy versus the same independent variable. The AE energy for tube-enhanced mortars not only maximizes at the 0.4 wt.% tube loading previously found associated with the dramatic 479% increase in fracture energy, but the relative increase in AE energy at the specific loading is almost identical to that of fracture energy. After that maximum, AE energy follows a decreasing trend with tube loading which is analogous to that of fracture energy. The AE energy released in micro-cracking phenomena during flexural testing of the mortars containing 0.4 wt.% nanotubes is 4 times higher than the value of control specimens, while corresponding improvements at concentrations higher than 0.4 wt.% were smaller. This finding independently confirms the previous claim that above a CNT loading threshold, side-effects such as nanotube agglomeration may impose adverse effects to the mechanical performance of the mortars. Such argumentation is fully compatible with previous findings that the advantageous effect of carbon nanotubes is limited to small concentrations, whereas less positive, or even adverse effects can be encountered at higher concentrations [67,68]. The trend of AE energy of GNP-modified mortars appears to reach a plateau at a concentration of 0.8 wt.%. The evidenced exceptional accuracy of the AE technique in validating the fracture response of the mortars, highlights it as capable of performing autonomous characterization of corresponding material systems and structures.

### 3.3. Electrical Resistivity

**Figure 10:**
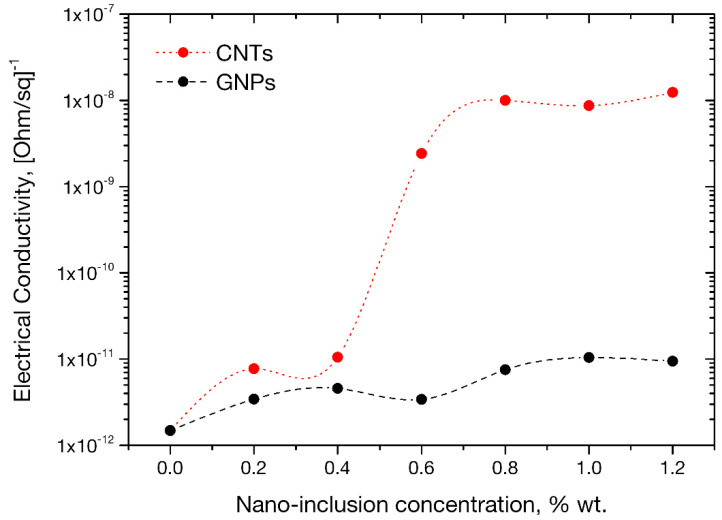
Surface electrical conductivity variation with CNT and GNP concentration.

The average electrical conductivity documented at each CNT/GNP concentration is plotted along with standard deviations in Figure 10. The documented trend highlights a dramatic dependence of nanotube presence in the electrical properties of the mortars with conductivity values appearing higher by 3 orders of magnitude from the reference value at a tube concentration of 0.6 wt.%, which correspondingly constitutes the electrical percolation threshold of the investigated material. At that concentration, the formation of a critical conductive network (CNTs in critical contact) enables a drastically higher electric flow throughout the mortar volume. Such percolation conditions in a multifunctional system impart damage sensing potential, hence also smartness, as required in contemporary applications. As observed in Figure 10, the electrical response of mortars enhanced with graphene nanoplatelets, does not follow a similar trend. Even at the highest concentration, the values of electrical conductivity remain at the same level as reference specimens, clearly indicating that the percolation threshold has not yet been reached. This finding can be undoubtedly attributed to nanoplatelets’ planar morphology, which, compared to their rod-like tube counterparts, requires higher concentrations for the formation of a critically conductive network across the material.

The documented enhancement of the mortars’ electrical conductivity by three orders of magnitude at the percolation threshold is the key requisite property towards the achievement of the piezoresistive effect in cement-based material, wherein electrical resistivity varies with applied strain. In turn, the effect endows strain and damage sensing abilities to the structural material, rendering it a smart, self-monitoring one, able to sense its own strain and damage (crack and damage formation and propagation) condition. Such strain and damage sensitivity of the nanocarbon-enhanced material compared to its pure cement counterpart, is of paramount usefulness towards its structural health monitoring, structural vibration control and load monitoring which eventually leads to more performant, durable and safer structures.

## 4. Conclusions

The current study reports on the effect of carbon nanotube and graphene nanoplatelet presence as nano-fillers at directly comparable loadings variable within 0–1.2 wt.% on the flexural, compressive, fracture mechanical and electrical properties of cement mortars targeted for contemporary applications with combined multifunctional potential and internal sensing abilities imparting smartness. Pure bending tests under four point bending configuration indicated that mortars enhanced with tube concentrations of 0.4 wt.%, exhibited optimum mechanical response. For GNP-enhanced mortars, the property improvement was by 21.52% higher than that offered by CNTs and was found maximum at a 1% nanoplatelet concentration. The compressive strengths of the two types of nano-modified mortars did not present significant improvements from control values. On the other hand, a remarkable improvement of 479% in fracture energy was measured for mortars containing 0.4% by weight of cement carbon nanotubes. Mortars enhanced with 1wt.%. GNPs exhibited dramatic, statistically consistent improvements, up to 870%, in fracture energy with respect to plain specimens, indicating an excellent potential of the material system for next generation applications with extreme fracture toughness demands. The particular finding is of increased significance for an inherently brittle material system such as cement. An exceptionally favorable comparison of AE and fracture energy data and trends with nano-filler concentration was observed which highlights a notable potential of the non-destructive technique in independently assessing the mechanical performance of cementitious materials reinforced at the nanoscale. Electrical measurements demonstrated the establishment of an electrical percolating CNT network in the mortars, at a tube concentration of ca. 0.5 wt.%. The increase in electrical conductivity by three orders of magnitude at that percolation threshold imparts piezoresistive properties to the mortars, rendering them smart, self-monitoring structural materials for contemporary applications with integrated structural health monitoring abilities. In GNP-enhanced mortars, no considerable electrical property improvements were recorded up to a concentration of 1.2 wt.%, indicating that the establishment of electrical percolation conditions in such mortars requires higher concentrations of the planar nanofiller. Further work on the sensing properties of the developed materials can reveal the extent of their strain and damage sensing potential towards exploitation in smart cement-based structural materials.

## Figures and Tables

**Table 1:** Main characteristics of multi-walled carbon nanotubes (MWCNTs).

Parameter	Value
Length	5–15 μm
Diameter	20–40 nm
Purity	≥97%
Ash content	≤0.2 wt.%
Special surface area	40–300 m^3^/g
Amorphous carbon content	≤3% wt.%

**Table 2:** Main characteristics of graphene nanoplatelets (GNPs).

Parameter	Value
Carbon content	>97% wt.%
Oxygen content	<0.6 wt.%
Sulphur content	<0.2 wt.%
Apparent density	60 g/L
pH	5–7
Lateral dimension	<10–15 μm
Thickness	<2–3 nm

**Table 3:** Values of coefficients hi for the calculation of conversion factor *k_d_* for d = 3 mm [65].

h_1_	h_2_	h_3_	h_4_
0.1037	104.1	105.1	0.01631

**Table 4:** Variation of mortars’ strengths with CNT and GNP concentration.

Concentration wt.% of Cement	Flexural Strength [MPa]		Compressive Strength [MPa]	
	CNTs	GNPs	CNTs	GNPs
0	6.03 ± 0.38		64.98 ± 2.56	
0.2	6.96 ± 0.8	4.73 ± 0.67	66.15 ± 1.07	74.10 ± 1.87
0.4	7.97 ± 0.05	5.12 ± 0.31	65.21 ± 0.81	76.77 ± 2.76
0.6	6.93 ± 0.34	7.46 ± 0.49	66.35 ± 3.53	75.83 ± 2.67
0.8	7.38 ± 0.18	7.58 ± 1.15	71.51 ± 1.85	78.45 ± 2.63
1.0	7.11 ± 0.61	9.57 ± 0.26	68.05 ± 2.92	81.70 ± 1.48
1.2	6.43 ± 0.67	9.30 ± 0.23	69.25 ± 4.35	59.8 ± 1.08

**Table 5:** Values of fracture energies of mortars originating from nano suspensions.

Concentration wt.% of Cement	Fracture Energy [N/m (J/m2)]	
	CNTs	GNPs
0	4.51 ± 0.44	
0.2	8.14 ± 0.74	18.73 ± 0.91
0.4	26.15 ± 1.08	33.54 ± 1.81
0.6	12.00 ± 1.23	37.66 ± 1.99
0.8	9.25 ± 0.76	42.99 ± 1.72
1.0	10.20 ± 0.82	43.76 ± 1.87
1.2	9.85 ± 0.83	41.64 ± 2.08

## Data Availability

Not applicable.
